# The Politics and Power of Populism: A Response to the Recent Commentaries

**DOI:** 10.15171/ijhpm.2017.118

**Published:** 2017-10-03

**Authors:** Ewen Speed, Russell Mannion

**Affiliations:** ^1^School of Health and Social Care, University of Essex, Colchester, UK.; ^2^Health Services Management Center, University of Birmingham, Birmingham, UK.

We would like to thank all of the authors who contributed their ideas in response to our editorial. Their commentaries have helped to shape and shift our thinking in this important but under researched area of contemporary healthcare politics and policy. We wrote the initial editorial^1^ at a time when many of the ideas we were grappling with were still quite nascent and not fully formed. Subsequently, many of the issues we introduced have become more prominent in the political and healthcare landscapes, others less so, (we characterise this as the politics of populism). Indeed, the only constant appears to be the continued shift towards a populist turn in contemporary western politics. The ubiquity of populism, coupled with the inherent difficulty of tracking the politics of populism has led us to further develop and nuance our initial argument (with the help of insights offered by the various respondents). In sharpening our thinking, it appears that a much more fruitful approach to the study of populism would to be explore what sorts of actions it allows (or disallows) and by whom? In this framing, the study of populism becomes an empirical endeavour with populism viewed as a performative instrument,^2^ as something that policy happens through, and the empirical concern becomes one of identifying and exploring the specific types of performances of politics that are played out through a variety of populist frames.

For example, McKee and Stuckler^3^ take up the notion of othering and difference, thereby providing novel insight into the processes involved in identifying the social groups that constitute ‘the people,’ and those that do not. They make clear that the current swing to populism is only the most recent in a long line of political appeals to identifying ‘enemies of the people,’ from the time of ancient Rome, through the French and Russian revolutions through to Nazi Germany. In a clever twist, after Ibsen’s Dr Stockman, they make the case for the need for experts to speak truth to power, even if this makes them an ‘enemy of the people.’ That is to say, going against the majority will of the populous. The imperative to identify unseen patterns of disease, describe hidden inequalities and give voice to those who are marginalised must outweigh any prevailing rejection of expertise. But how to do this, how to speak out against these prevailing populist mores? It is in situations such as this where the performative utility of populism comes to the fore, as it functions to pre-judge and critique the role of expertise, disavowing it before it is even able to engage in the debate. Thus, the empirical imperative must be one that understands the ways in the performance of expertise and anti-expertise are played out, and to subsequently develop ways of countering these moves. Providing more specific expertise about the particular case in health policy plays right into the hands of the anti-experts.

Schrecker^4^ argues that the problem is not populism per se, but rather the very distinctive form of populism currently being appropriated by politicians on the right, coupled with a very real failure of politicians on the left to develop compelling counternarratives. Shcrecker points out that the crux of the populist problem is how it serves as a vehicle for the transnational capitalist class to misdirect the identification of threats to the health and wellbeing of populations left behind by neoliberal economic integration. This talk of misdirection speaks to questions we initially raised in terms of questions of post-truth and fake news. The way in which these concepts operate is to splinter or shatter the idea that there is one version of events or absolute truth. They do not operate so much as to attain hegemony over other interpretations, rather they seek to operate in tandem, alongside other representations, such that it becomes possible (with a post-modern flourish) to argue any number of representations might be true. The intention is to challenge the narrative, and once this challenge becomes ‘true,’ then any other counter-narratives also becomes possible. This is the very process that needs to be unmasked and addressed, and in our opinion wasting time identifying ‘successful’ or ‘convincing’ counter-narratives is just pouring oil on the post-truth fire. Similarly, Powell^5^ challenges our use of the notion of post-truth, stating that politics operates in a way where the relation between political rhetoric and what is ‘true’ has always been somewhat loosely construed. Again, we would agree with this. But, the point is that politicians, at this particular point in history, are utilising notions of fake news and post-truth politics – their authenticity is not what is of analytical interest, rather it is the ubiquity of these forms of discourse which piques our curiosity. What is to be gained, now, at this point in time, by the deployment of these populist tropes?

What is most compelling from Powell’s commentary for us, is the unpacking of the complexity inherent in making sense of the relation between populism and health policy. He states that it is difficult (impossible?) to causally link different types of populism (left, right, centrist) with impacts on health policy. We would certainly agree with this point. Powell then moves on to offer a consideration of the cases we presented, demonstrating a range of complexity inherent in making sense of the policy issues as populist. He offers a range of sources of evidence which demonstrate the clear disparity (perhaps even untruth) between the evidence available on any of these issues and the populist framing of those self-same issues. In this context Powell does us a service, demonstrating unequivocally how the rhetoric of populism is almost completely undermined by any hard interrogation of the facts. In large part we agree with Powell’s characterisation of the complexity of these issues, but this brings us back to the initial question, why populism, why now and in addition, how are the political elites accomplishing this populist turn? Powell demonstrates the clear disjuncture between populist politics and the ‘real world’ of evidence, so how is it that populist politics appears to be trumping (no pun intended) ‘real world evidence.’ This presents the imperative (and very real) need to understand the logic, power and appeal of populism within contemporary (healthcare) politics

De Cleen^6^ offers a range of important insights into the theoretical frame that we propose. He is convincing in stating that there is far more complexity to the dynamics of populism than we allow in our initial piece. De Cleen offers a number of additional conceptual elements or layers that he stresses need to be considered to avoid falling into a naïve reading of populism. For example, how the people/elite axis intersects with the in/out membership axis, could offer insight into how exclusionary (or inclusionary) different types of populism might be in a healthcare context. Like Powell, De Cleen asserts that populism can be both of the right or the left, and that there is a clear need to pay attention to the political orientation of the populist politics. We concur, but for us the question is about how this is best achieved. Explicit attempts to undermine expertise, also function to undermine critique, (you are either for us or against us) such that the political bent of any political activity might be subsumed under claims to be for the people, against the elite. In this context expertise becomes a shorthand for any oppositional view. De Cleen is right to assert that expertise is neither objective nor value free, and it was not our intention to suggest it was, our concern was with pointing out how populism might work to silence critique, and an anti-expertise focus is, we feel, a central part of that silencing. De Cleen’s analysis of our paper offers important insights which we are grateful for, but again, we feel these need to be worked through in terms of how they enable or allow populist logics to perform through healthcare policy.

Taggart^7^ offers a different slant, one predicated on responses to populism. It offers useful and positive solutions, predicated on the principles of community psychology. However, Taggart’s view almost takes us too far away from the performance of populism, requiring us to fashion a response before we have applied our critique. Certainly, anything that encourages democratic exchange and engagement offers a powerful counterpoint to the worst excesses of post-truth populism.

Halikiopoulou^8^ offers important insight into the dynamic process of populism, and raises questions about the study of left and right wing populism and the need to distinguish between them. In part we agree, but more interesting for us is the way in which Halikiopoulou’s argument sets up the possibility of distinct processes, such as welfare chauvinism, nativist policy making and such like from other discreet political processes. Halikiopoulou identifies (correctly we feel) what the import of these processes are, and it is the need to understand populism as a set of political practices that we feel is key in setting the populist politics research agenda for health policy analysts and social scientists in the immediate future.

## Ethical issues

Not applicable.

## Competing interests

Authors declare that they have no competing interests.

## Authors’ contributions

Both authors contributed equally to the writing of this article.

## Authors’ affiliations

^1^School of Health and Social Care, University of Essex, Colchester, UK. ^2^Health Services Management Center, University of Birmingham, Birmingham, UK.
